# Feature tracking derived longitudinal and circumferential myocardial strain abnormalities in clinical myocarditis

**DOI:** 10.1186/1532-429X-17-S1-P321

**Published:** 2015-02-03

**Authors:** Justin D Weigand, James Nielsen, Partho Sengupta, Javier Sanz, Shubhika Srivastava, Santosh Uppu

**Affiliations:** 1 Pediatric Cardiology, Mount Sinai Hospital / Icahn School of Medicine, New York, NY, USA; 2 Division of Pediatric Cardiology, Stony Brook Children's Hospital, Stony Brook, NY, USA; 3 Department of Cardiology, Mount Sinai School of Medicine, New York, NY, USA

## Background

Cardiac magnetic resonance imaging (CMR) is frequently used to assess myocardial involvement to confirm diagnosis of myocarditis. CMR techniques employ T2- weighted, early and late gadolinium enhancement (LGE), with two positive sequences widely considered diagnostic for myocardial inflammation. However, these techniques are qualitative and subjective, thus prone to interpretation error. Feature tracking (FT) allows quantitative segmental myocardial strain analysis using traditional cine CMR images. Our hypothesis was that left ventricular (LV) segmental strain would be a more sensitive indicator for myocardial involvement in patients with clinical myocarditis. Regional strain was compared to presence of LGE as a measure of myocardial involvement.

## Methods

Patients with clinical diagnosis of myocarditis and structurally normal hearts who underwent CMR at our institution from 2004-2014 were studied retrospectively. Consecutive healthy young adults with normal cardiac anatomy, function and absent LGE were selected as controls. FT was performed on CMR cine images; longitudinal strain analysis was performed in four chamber (4C) and two chamber (2C) images, circumferential strain was calculated at basal, midventricular (mid), and apical levels. Global and segmental strain data was compared to troponin leak and presence of LGE. Sensitivity, specificity, and criterion values of strain were determined by comparison to controls. Descriptive statistics, t-tests, logistic regression analysis, and receiver operator characteristic curves were utilized.

## Results

Twenty-nine myocarditis patients and nineteen controls were analyzed by FT. All myocarditis patients demonstrated abnormal myocardial strain in at least one longitudinal or circumferential distribution including patients in whom LGE was negative. Twenty of the 29 myocarditis patients with normal ejection fraction were included in the final analysis. Myocarditis patients had significantly diminished longitudinal 4C, longitudinal 2C, global circumferential, circumferential basal, mid, and apical strain when compared with normal controls (Table 1). Multivariate logistic regression analysis identified longitudinal 4C strain as the most predictive for troponin leak and LGE (p= <0.01 respectively). Longitudinal 4C and global circumferential strain demonstrates good sensitivity and specificity for troponin leak and LGE. Circumferential basal and mid ventricular strain demonstrates excellent sensitivity for troponin leak and LGE. Circumferential apical strain has good specificity for troponin leak (Table 2).

**Table 1:** Summary statistics

	Control	Myocarditis with Normal Function	p-value
N	19	20	

Median Age (years, IQR)	19 (18-20)	18 (17-18.6)	

Male:Female	13:6	18:2	

Mean BSA (m <2>)	1.9	2	0.36

Elevated Troponin	0	19	< 0.01

Mean LV EF (%)	61	62	0.32

(+) LGE	0	18	< 0.01

Longitudinal 4C Strain	-18.4 ± 3.9	-14.6 ± 3.5	< 0.01

Longitudinal 2C Strain	-18.7 ± 4.9	-15.2 ± 5.1	0.03

Global Circumferential Strain	-22.7 ± 3.2	-17.9 ± 3.5	< 0.01

Circumferential Basal Strain	-22.8 ± 4.5	-18.2 ± 4.6	< 0.01

Circumferential Mid-ventricular Strain	-20.5 ± 3.6	-17.4 ± 2.3	< 0.01

Circumferential Apical Strain	-24.7 ± 5.0	-18.1 ± 5.6	< 0.01

Mean ± standard deviation unless as indicated

**Table 2:** Receiver operating characteristic curves for elevated troponin and late gadolinium enhancement

Strain	Elevated Troponin					Late Gadolinium Enhancement				
	
	AUC	Strain Criterion Value	Sensitivity (%)	Specificity (%)	p-value	AUC	Strain Criteria Value	Sensitivity (%)	Specificity (%)	p-value
Longitudinal 4C	0.81	-15.83	83	84	< 0.01	0.79	-15.5	72	86	< 0.01

Longitudinal 2C	0.69	-14.4	56	84	0.03	0.72	-14.4	61	86	0.01

Global Circumferential	0.86	-20.8	83	74	< 0.01	0.83	-20.5	83	76	< 0.01

Circumferential Basal	0.77	-24.2	100	58	< 0.01	0.76	-24.2	100	52	< 0.01

Circumferential Mid-ventricular	0.77	-20.8	94	63	< 0.01	0.75	-19.8	89	67	< 0.01

Circumferential Apical	0.81	-21.1	67	84	< 0.01	0.80	-21.8	83	67	< 0.01

## Conclusions

FT derived longitudinal and circumferential strain is more sensitive and specific in identifying myocardial involvement. FT can be a useful adjunct to traditional CMR techniques as an objective measure of myocardial involvement especially in patients with myocarditis and normal LV function. Feature tracking may prove advantageous in cases of clinical myocarditis where traditional CMR techniques are inconclusive.

## Funding

N/A.

